# Nonoperative Treatment of Adult Spinal Deformity: A Comprehensive Narrative Review

**DOI:** 10.3390/jcm14248864

**Published:** 2025-12-15

**Authors:** Christos G. Zlatanos, Mohamed A. Hassanin, Ahmed Aly, Khalid M. Salem, Nasir A. Quraishi

**Affiliations:** 1 Centre for Spinal Studies and Surgery, Queens Medical Centre, Nottingham University Hospitals NHS Trust, Nottingham NG7 2UH, UK; mmghafoorhassanien@aun.edu.eg (M.A.H.); khalid.salem2@nhs.net (K.M.S.); 2 Department of Orthopaedics and Trauma Surgery, Assiut University Hospitals, Assiut 71515, Egypt; 3 Neurosurgery Department, Faculty of Medicine, Aswan University, Aswan 81528, Egypt; ahmedaleem2000@gmail.com

**Keywords:** adult spinal deformity, non-operative treatment, spinal deformity outcomes, conservative management

## Abstract

**Background/Objectives**: Adult spinal deformity (ASD) is increasingly prevalent due to an ageing population and is associated with significant pain, disability, and reduced quality of life. While surgery is often considered for severe deformities, many patients are either unsuitable for major corrective procedures or prefer conservative care. This narrative review synthesizes the current evidence on nonoperative management strategies for ASD. **Methods**: A literature search on the PubMed and Cochrane databases identified relevant studies published up to 25 October 2025. Medical Subject Headings and keywords related to nonsurgical ASD management were used. Eligible studies included nonsurgical series with a minimum of 12 months’ follow-up, while case reports were excluded. **Results**: Seven studies met our inclusion criteria: three on bracing, three on physiotherapy and combined physical and cognitive rehabilitation programmes, and one on transforaminal epidural steroid injections (ESIs). Bracing was effective in slowing the curve progression rate. One study showed that the progression rate decreased from 1.47°/year to 0.24° for degenerative scoliosis (*p* < 0.0001) and from 0.70°/year to 0.24° for idiopathic scoliosis (*p* = 0.03). Another study showed that there was no statistically significant difference in the Cobb angle or anticipated worsening when comparing the initial measurement with the final control after treatment (*p* = 0.973). Finally, a third study reported reduced back pain, with Roland–Morris scores improving from 3.3 to 2.0 (*p* < 0.001) at 18 months. Physiotherapy and multidisciplinary rehabilitation programmes appeared to be effective in significantly reducing pain and disability levels. One study found that Oswestry Disability Index (ODI) scores improved from 39.5 to 31.8 (*p* < 0.001), while back pain, measured using the Numeric Pain Rating Scale (NPRS), improved from 58.4 to 42.1 (*p* < 0.001), with 51% achieving minimal clinically important change (MCIC). Another study reported ODI reductions from 38 to 17.6 and pain scores from 6.5 to 2.2 (*p* < 0.001), while in a third study, the “Koshimagari Exercise” programme yielded MCIDs in the ODI for 42% of patients. Finally, ESIs provided significant pain relief for at least a month in over half of the patients with degenerative scoliosis and radiculopathy, with diminishing effects throughout the first 2 years. More specifically, 37.2% of patients had a successful outcome at one year post-injection and 27.3% at 2 years (*p* < 0.01). **Conclusions**: Our study suggests that bracing, physiotherapy, and multidisciplinary rehabilitation programmes, as well as ESIs, can serve as effective short term alternatives for patients with ASD who are either unsuitable for surgery or do not wish to pursue it. As such, this review provides valuable evidence-based insights that can guide clinicians in developing a treatment plan and lay the foundations for establishing a novel pathway for this specific subgroup of patients with ASD.

## 1. Introduction

Adult spinal deformity (ASD) represents a growing public health concern, which is largely attributable to the ageing global population. It is estimated that more than 15% of adults in the United States have scoliosis, with a prevalence of up to 68% in people above 60 years of age [1,2], while the total number of ASD surgeries has increased significantly in the last decade [3].

There is overwhelming evidence from systematic reviews and meta-analyses that surgical treatment is superior to nonoperative management in terms of pain and disability levels, as well as quality of life and overall clinical outcomes [4,5]. Surgery, however, not only comes with a complication rate as high as 80% in some cases [6] but is also associated with a substantial financial burden, with total hospital costs averaging USD 120,394 per patient and significantly increasing after primary surgery [7]. Nonsurgical treatments, on the other hand, are an alternative option for patients who are not surgical candidates or for those who have refused surgical intervention; these modalities include bracing, physical therapy along with cognitive–behavioural rehabilitation programmes, and pain management interventions including epidural steroid injections (ESIs).

Despite the widespread use of these conservative treatment modalities, the current evidence supporting their effectiveness is fragmented and limited. Much of the existing research is derived from small case series, heterogeneous patient populations, and varying treatment protocols, making it challenging to draw definitive conclusions about the optimal nonoperative management strategies for ASD [5].

To address these gaps in knowledge, we conducted a comprehensive narrative review, aiming to evaluate the current evidence on nonoperative treatment strategies for ASD to provide a comprehensive and evidence-based overview of their efficacy, limitations, and role in the management of ASD.

## 2. Materials and Methods

A comprehensive search was conducted across two major electronic databases: PubMed and Cochrane. The search included studies published from the inception of each database until 25 October 2025. The search strategy employed a combination of Medical Subject Heading (MeSH) terms and keywords to identify relevant studies, as follows:

“((non operative) OR (conservative) OR (non surgical)) AND (management OR treatment) AND ((adult spinal deformity) OR (adult scoliosis) OR (degenerative scoliosis)) NOT adolescent NOT neuromuscular NOT syndrome NOT ankylosing spondylitis NOT congenital NOT Chiari NOT spina bifida NOT vascular NOT tumour NOT tumour NOT infection NOT trauma”.

All searches were limited to human, English-language studies with a minimum follow-up of 12 months. Publications that did not contain original data, review articles, case reports, and letters were excluded.

The following data was extracted from the included articles (Table 1):

- Study characteristics: authors, publication year, study design, and sample size.
- Patient characteristics: age, sex, and diagnosis (e.g., degenerative scoliosis, adult idiopathic scoliosis).
- Type of nonoperative treatment (e.g., bracing, rehabilitation programmes, ESIs).
- Outcomes: ODI, NRS (Numeric Rating Scale), SF36 (36-Item Short Form Survey), SRS-22 (Scoliosis Research Society 22-item questionnaire), Roland and Morris VRS (Verbal Rating Scale), and curve progression (Cobb angle).

## 3. Results

Our initial search identified 860 articles. After the removal of duplicates, all records were screened for eligibility based on their titles and abstracts. This step excluded 828 records that did not meet the predefined inclusion criteria. Full-text assessments were performed on 32 articles to evaluate their relevance and methodological quality. Following this evaluation, seven studies meeting the inclusion criteria were included in the final review. These are summarized in Table 1.

### 3.1. Bracing

Three studies in this review evaluated the role of bracing in the management of ASD. The first study, a prospective trial with 158 patients conducted by de Mauroy et al. [8], utilized custom-moulded lumbar–sacral orthoses. Patients were treated initially with a plaster cast made in a specific standing frame for 24 h/day for 3 weeks, followed by application of a custom-moulded rigid brace for at least 4 h/day for a minimum of 6 months. The mean age was 56 years (SD = 13), the baseline mean Cobb angle was 40 degrees, and the follow-up period was 8 years. Outcome measures included Cobb angle progression, rib hump measured in mm, occipital axis, and T1 plumb line. Comparing means at the beginning and last control, the study reported no significant difference in Cobb angle (*p* = 0.973), indicating stabilization of the curve, sagittal balance (*p* = 0.231), and rib hump (*p* = 0.073), while the coronal balance improved significantly (*p* = 0.008).

In the second study, a retrospective analysis by Palazzo et al. [9], 38 participants with adult idiopathic and degenerative scoliosis were treated with custom-moulded lumbar–sacral orthoses for a minimum of 6 h/day and followed for 8.7 ± 3.3 years after bracing. The mean baseline Cobb angle was 49.6° (SD = 17.7°). In patients with degenerative scoliosis, there was a drop in the Cobb angle progression rate from 1.47 degrees/year ± 0.83 before to 0.24 ± 0.43 after bracing (*p* < 0.0001), while for idiopathic scoliosis, the rate dropped from 0.70 degrees/year ± 0.06 before to 0.24 ± 0.43 after bracing (*p* = 0.03).

Finally, Weiss et al. [10] evaluated the efficacy of a sagittal realignment brace (Physio-Logic™ brace) in 67 patients with back pain associated with adult idiopathic or degenerative scoliosis, as well as hyper-kyphosis. Patients were instructed to wear the brace for a minimum of 20 h per day during the initial six months, after which brace wearing times were set by the individual patient, depending on their residual pain levels. The average pain intensity, measured using the Roland and Morris Verbal Rating Scale (VRS; five-point scale), decreased from 3.3 at baseline to 2.0 at the 18-month follow-up (*p* < 0.001), indicating a statistically significant reduction in back pain.

### 3.2. Physical Therapy and Combined Physical and Psychological Programmes

Three studies examined the role of physical therapy and combined physical and psychological programmes in the treatment of ASD. Hoevenaars et al. [11] conducted a retrospective analysis of 320 patients, 80 with ASD (both adult idiopathic and degenerative scoliosis) and 240 without ASD but with chronic low-back pain, who were treated with a combined physical and psychological rehabilitation programme over a 12-month period. In the ASD cohort, the Oswestry Disability Index (ODI) improved from 39.5 (±12.0) at baseline to 31.8 (±16.5) at one-year follow-up (*p* < 0.001).

Back pain intensity, as measured by the Numeric Pain Rating Scale (NPRS), improved from 58.4 (+/−19.1) to 42.1 (+/−28.3) (*p* < 0.001). The mean physical component of the 36-Item Short Form Survey (SF36) improved from 39.8 (±14.4) to 56.6 (±20.3), while the mean mental health component improved from 55.7 (±18.3) to 67.2 (±20.1) (*p* < 0.001). Mean self-efficacy, measured by the Pain Self-Efficacy Questionnaire (PSEQ), improved from 30.1 (±10.5) to 43.4 (±13.3) (*p* < 0.001), while 51% of the ASD patients reached minimal clinically important changes (MCICs) and 33% reached a Patient-Acceptable Symptom State (PASS).

The second study, conducted by Monticone et al. [12], was a randomized controlled trial of 130 adults with idiopathic scoliosis and a mean baseline curve of 28° who were followed for 12 months. The treatment group (n = 65) underwent a 20-week rehabilitation programme combining active self-correction, task-oriented exercises, and cognitive–behavioural therapy. The control group (n = 65) received general physiotherapy (mobilization, stretching, strengthening). In the treatment group, there were significant drops in the ODI from 38 to 17.6 (*p* < 0.001), numeric rating scale (NRS) from 6.5 to 2.2 (*p* < 0.001), Tampa Scale from 31.5 to 18.8 (*p* < 0.001), and Pain Catastrophizing scale from 27.6 to 15.9 (*p* < 0.001). With regard to complications, only minor adverse effects were reported, including transient worsening of pain (experimental group: n = 5; control group: n = 3) and mood disorders (experimental group: n = 2; control group: n = 4) that were easily managed by symptomatic drugs and brief periods of rest.

Finally, Taniwaki et al. [13] assessed the impact of “Koshimagari Exercise”, a self-exercise programme for ASD patients. The programme combined weekly physiotherapist-led sessions with home-based exercises performed three times per week with a minimum follow-up of 12 months, focusing on flexibility, functional improvement, trunk and back muscle strengthening, motor learning, and pain control. The study reported that 42% of participants achieved a minimal clinically important difference (MCID) in the Oswestry Disability Index (ODI), along with significant improvements in EQ-5D and Visual Analogue Scale (VAS) scores for low-back pain from baseline (*p* < 0.001).

### 3.3. Epidural Steroid Injections

There was only one study examining the role of transforaminal epidural steroid injections. Cooper et al. [14] retrospectively studied the role of transforaminal epidural steroid injection (TFESI) in 61 patients with degenerative lumbar scoliosis with spinal stenosis and radiculopathy. A successful outcome was defined as a patient who was both satisfied with the results of the intervention (North American Spine Society (NASS) satisfaction scale 1 or 2) and experienced at least a two-point improvement in NRS, Summary Pain, and Summary Function scores, as measured using the adapted Stucki questionnaire. In this study, 37.2% of patients had a successful outcome at one year post-injection, and 27.3% at 2 years (*p* < 0.01).

## 4. Discussion

It is well established in the literature that surgery can provide superior outcomes compared to conservative treatment in patients with adult spinal deformity (ASD) [4,5,15]. Nevertheless, the question remains what the optimal treatment is for patients who are either unfit for surgery or who do not wish to pursue it. This narrative review provides an insight into the role of nonoperative options in managing adult spinal deformity (ASD), suggesting that modalities such as bracing, physiotherapy and multidisciplinary rehabilitation programmes, and steroid epidural injections can be meaningful alternatives providing short-term relief for this patient group.

Operative treatment can significantly reduce disability and pain, while at the same time improving clinical outcomes, although it is associated with a complication rate of up to 80.3% in some studies (Choi et al. [4], Jia et al. [5]). Similarly, Smith et al. [15] reported substantial improvements in health-related quality of life (HRQoL) of surgical patients at a minimum two-year follow-up, with 71.5% of them experiencing at least one complication.

In a nonrandomized clinical trial published in 2025 with an 8-year follow-up, Smith et al. [16] demonstrated that operative treatment for adult patients with lumbar scoliosis resulted in significantly greater clinical improvements than nonoperative management at 2-, 5-, and 8-year follow-up, with no evidence of deterioration—suggesting the long-term durability of surgical treatment. In contrast, Finoco et al. [17] demonstrated in their recent multicentre study that both operative and nonoperative treatments were associated with clinical improvement at the 2-year follow-up in adults with degenerative scoliosis. The physical component scores of the SF-12 (PCS) improved in both groups, with no significant differences being observed between surgical and nonsurgical patients. Similarly, VAS, ODI, and SRS scores did not differ significantly between the two groups.

Bracing has historically been utilized for younger populations with scoliosis, yet its role in ASD remains an area of ongoing research. The three studies included in this review [8,9,10] examining bracing demonstrated promising results in stabilizing the Cobb angle and deformity progression over time, especially in patients with milder deformity, as well as in reducing the severity of back pain. In their systematic review conducted in 2020, McAviney et al. [18] concluded that there was evidence, albeit from low-quality studies, to suggest that treatment with a brace may have a positive short- to medium-term influence on pain and function in adults with degenerative or idiopathic scoliosis. However, limitations remain regarding patient compliance, discomfort, and the long-term effects of bracing on pain and quality of life.

With regard to physiotherapy and combined physical and psychological programmes, it was found in this review that combining targeted physical therapy, postural retraining, and cognitive–behavioural strategies may help mitigate pain and improve overall quality of life [11,12]. Hoevenaars et al. [11] found that the ASD cohort improved following rehabilitation, and that these improvements were maintained for at least one year. The improved outcomes in ASD patients appear clinically equivalent to the non-ASD cohort, without any statistical difference between the two cohorts with respect to outcome parameters at any time point. This is in contrast with studies reporting that nonsurgical treatment does not demonstrate sustained changes in the quality of life of ASD patients [19]. Moreover, combined motor and cognitive rehabilitation seems to be superior to general physiotherapy in reducing disability for adults with idiopathic scoliosis, as Monticone et al. [12] found in their study. Similarly, Taniwaki et al. [13] showed that 42% of the patients participating in the “Koshimagari Exercise” programme achieved a minimum clinically important difference (MCID) in the ODI, along with statistically significant improvements in EQ-5D and VAS scores for low-back pain from baseline.

Finally, with regard to the role of epidural steroid injections, only one study matched our criteria and was included in this review. Cooper et al. [14] concluded that transforaminal epidural steroid injections were effective for patients with degenerative lumbar scoliosis and radiculopathy, providing symptom relief for up to two years in more than a quarter of patients (27.3%). Other studies with shorter follow-up periods have also reported similar findings [20].

There are several limitations associated with this narrative review. Firstly, the heterogeneity of the studies and the lack of standardized outcome measures make it difficult to draw definitive conclusions, whereas much of the existing literature is based on observational designs with inherent methodological constraints that should be considered when interpreting the results. Additionally, long-term data on nonoperative management is still limited, raising questions about the sustainability of treatment effects. Furthermore, it is worth noting that only the study by Monticone et al. [12] explicitly reported complications and adverse effects, despite the well-documented risks associated with both bracing and epidural injections—such as brace intolerance, skin irritation, and injection-related complications, including dural puncture or infections. Finally, with regard to ESIs, which are generally considered short- to medium-term pain management tools, we acknowledge that imposing a 12-month cutoff criterion may have led to the exclusion of several relevant studies.

## 5. Conclusions

In summary, this narrative review found that nonoperative treatment strategies, including bracing, physiotherapy and multidisciplinary rehabilitation programmes, and steroid epidural injections, may provide meaningful short term benefits in the management of ASD. The reviewed studies consistently reported positive functional outcomes associated with nonoperative treatments, with improved ODI scores in most studies, indicating enhanced functional capacity and quality of life, while a reduction in pain severity was a commonly observed benefit.

While the current evidence is limited by methodological constraints and varying levels of risk of bias, these limitations do not detract from the clinical relevance of the findings. Instead, they highlight the need for more rigorous research to strengthen and expand upon the insights provided here. This study may therefore serve as a useful guide for clinicians managing ASD patients who are medically unfit for surgery or who prefer nonoperative care, and it could help lay the groundwork for developing standardized treatment protocols for this specific patient population. Nonetheless, further high-quality research is required to establish evidence-based guidelines and clarify the long-term outcomes of nonoperative strategies for ASD.

## Figures and Tables

**Table 1:** Summary of studies included.

Study	Methodology	Treatment	No. of Patients	Mean Age (Years)	Deformity Type	Follow-Up Period	Mean Baseline Cobb Angle	**Outcome Measures**	**Results**
de Mauroy et al. (2016) [8]	Prospective	Bracing	158	56, SD = 13	Not specified	8 years	40°.	Cobb angle	No statistically significant difference when comparing the initial measurement with the final control after treatment (*p* = 0.973).
Palazzo et al. (2017) [9]	Retrospective	Bracing	38	61.3 ± 8.2 at the moment of bracing	Adult idiopathic and degenerative scoliosis	22.0 ± 11.1 years before bracing and 8.7 ± 3.3 years after bracing	49.6° ± 17.7°.	Cobb angle	Degenerative scoliosis: progression rate dropped from 1.47°/year ± 0.83 before to 0.24 ± 0.43 after bracing (*p* < 0.0001). Idiopathic scoliosis: rate dropped from 0.70°/year ± 0.06 before to 0.24 ± 0.43 after bracing (*p* = 0.03).
Weiss et al. (2009) [10]	Prospective	Bracing	67	Not available	Adult idiopathic and degenerative scoliosis, hyper- kyphosis	18 months	41°.	Roland and Morris Verbal Rating Scale (VRS)	Roland and Morris Verbal Rating Scale (VRS) improved from 3.3 before treatment to 2.0 (*p* < 0.001) at 18-month follow-up.
Hoevenaars, E.H. et al. (2022) [11]	Retrospective	Combined physical and psychological programme	320 (80 ASD plus 240 non-ASD	50.9 (±14.1) years (ASD cohort)	Adult idiopathic and degenerative scoliosis	12 months	21.4° (±9.4°) (ASD cohort).	ODI, NPRS	ODI improved from 39.5 (±12.0) at baseline to 31.8 (±16.5) at one-year follow-up (*p* < 0.001). NPRS improved from 58.4 (±19.1) to 42.1 (±28.3), *p* < 0.001.
Monticone, M. et al. (2016) [12]	Randomized controlled trial	Combined physical and psychological programme	130	51.6 (treatment group) and 51.7 (control)	Idiopathic scoliosis	12 months	28.2° (treatment group); 27.5° (control group).	ODI, NPRS, Cobb angle	ODI improved from 38 to 17.6 (*p* < 0.001), NPRS improved from 6.5 to 2.2 (*p* < 0.001). Cobb angle: no statistically significant difference (*p* = 0.074).
Taniwaki et al. (2025) [13]	Prospective multicentre	Physiotherapy—“Koshimagari Exercise”	98	73.0 (50–80)	Not available	12 months	Thoracolumbar: 12.5° (MCID-Achieved group); 14.1° (Non-achieved group). Lumbar: 18.9° (MCID-Achieved group); 18.3° (Non-achieved group).	ODI, EuroQol-5 Dimensions (EQ-5D), Visual Analogue Scale (VAS) for back pain	42% of patients achieved a minimum clinically important difference (MCID) in ODI and improvement in EQ-5D scores and Visual Analogue Scale (VAS) for low-back pain from baseline (*p* < 0.001).
Cooper, G. et al. (2004) [14]	Retrospective	Transforaminal ESI	61	68.6	Degenerative scoliosis	24 months	Unknown.	NPRS	37.2% of patients had a successful outcome (at least a 2-point improvement in NPRS) at one year post-injection, and 27.3% at 2 years (*p* < 0.01).

## Data Availability

All data generated or analyzed in this study are included in this published article.

## Abbreviations

The following abbreviations are used in this manuscript:

ASD	Adult Spinal Deformity
ESIs	Epidural Steroid Injections
ODI	Oswestry Disability Index
NPRS	Numeric Pain Rating Scale
MCIC	Minimal Clinically Important Change
MeSH	Medical Subject Headings
SF36	36-Item Short Form Survey
NRS	Numeric rating scale
SRS-22	Scoliosis Research Society 22-item questionnaire
VRS	Verbal Rating Scale
VAS	Visual Analogue Scale
SD	Standard Deviation
EQ-5D	EuroQol Five Dimension
PSEQ	Pain Self-Efficacy Questionnaire
PASS	Patient Acceptable Symptom State
TFESI	Transforaminal Epidural Steroid Injection
NASS	North American Spine Society
HRQoL	Health-related quality of life
